# Efficient Classification of ECG Images Using a Lightweight CNN with Attention Module and IoT

**DOI:** 10.3390/s23187697

**Published:** 2023-09-06

**Authors:** Tariq Sadad, Mejdl Safran, Inayat Khan, Sultan Alfarhood, Razaullah Khan, Imran Ashraf

**Affiliations:** 1 Department of Computer Science, University of Engineering & Technology, Mardan 23200, Pakistan; tariqsadad@uetmardan.edu.pk (T.S.); inayatkhan@uetmardan.edu.pk (I.K.); razaullah@uetmardan.edu.pk (R.K.); 2 Department of Computer Science, College of Computer and Information Sciences, King Saud University, P.O. Box 51178, Riyadh 11543, Saudi Arabia; sultanf@ksu.edu.sa; 3 Department of Information and Communication Engineering, Yeungnam University, Gyeongsan 38541, Republic of Korea; imranashraf@ynu.ac.kr

**Keywords:** cardiovascular diseases (CVDs), CNN, ECG, deep learning, IoT

## Abstract

Cardiac disorders are a leading cause of global casualties, emphasizing the need for the initial diagnosis and prevention of cardiovascular diseases (CVDs). Electrocardiogram (ECG) procedures are highly recommended as they provide crucial cardiology information. Telemedicine offers an opportunity to provide low-cost tools and widespread availability for CVD management. In this research, we proposed an IoT-based monitoring and detection system for cardiac patients, employing a two-stage approach. In the initial stage, we used a routing protocol that combines routing by energy and link quality (REL) with dynamic source routing (DSR) to efficiently collect data on an IoT healthcare platform. The second stage involves the classification of ECG images using hybrid-based deep features. Our classification system utilizes the “ECG Images dataset of Cardiac Patients”, comprising 12-lead ECG images with four distinct categories: abnormal heartbeat, myocardial infarction (MI), previous history of MI, and normal ECG. For feature extraction, we employed a lightweight CNN, which automatically extracts relevant ECG features. These features were further optimized through an attention module, which is the method’s main focus. The model achieved a remarkable accuracy of 98.39%. Our findings suggest that this system can effectively aid in the identification of cardiac disorders. The proposed approach combines IoT, deep learning, and efficient routing protocols, showcasing its potential for improving CVD diagnosis and management.

## 1. Introduction

**Figure 1:**
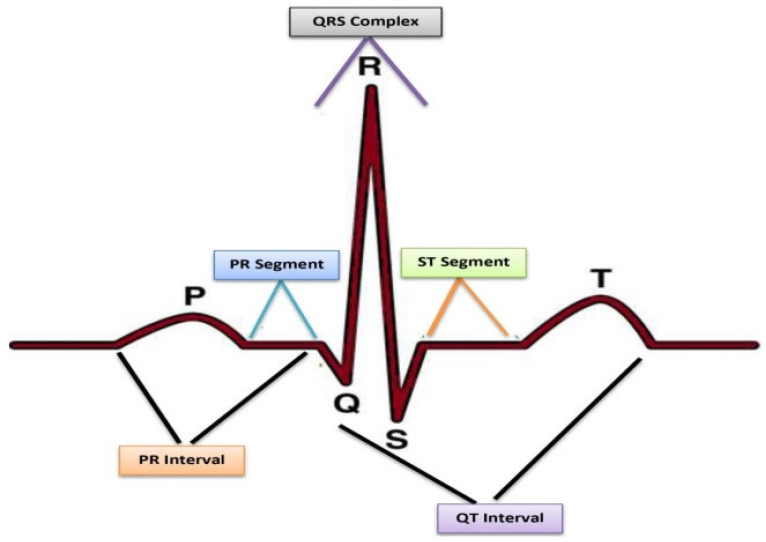
Illustration of normal ECG [4].

CVD is the leading chronic disease, according to the report of WHO (World Health Organization) [1]. Similarly, Centers for Disease Control and Prevention (CDC) reported that approximately 690,882 deaths occurred in the United States due to heart disease in 2020 [2]. However, CVD can be significantly diminished through early-stage and accurate diagnosis. In the modern world, advanced medical science has unveiled effective solutions for managing CVD problems, harnessing the power of information technology to handle diseases. The tools that are advised for cardiac diagnosis are ECG, Echo, ETT, blood testing, and angiography screening. Among these, ECG is the cheapest and most common diagnostic procedure of screening for heart disorders [3]. Furthermore, it is considered a standard tool for evaluating CVD in patients who live in remote areas. ECG is a non-stationary graphical signal used to detect the electrical movement of the heart by placing electrodes on the surface of the body. The graphical representation of normal ECG is demonstrated in Figure 1.

ECG signifies the probability of cardiac irregularities in their ST segments: usually, the elevations in ST segments, variations in segments, or flipping of T waves indicate some cardiac abnormalities [5].

The IoT refers to the interconnections of an assortment of smart devices, including mobile phones, laptops, and sensors [6,7,8,9]. One of the most compelling areas of application for the IoT is in the field of healthcare. The potential of the IoT has led to the development of various applications, such as remote health monitoring, fitness programs, elderly care, and the management of chronic ailments [10,11,12]. As a result, numerous medical devices, including diagnostic tools, imaging systems, and sensors, have transitioned into smart devices that serve as the foundation of the IoT. By utilizing IoT-based healthcare services, it is possible to reduce costs and improve user experience and quality of life.

Recently, applications for deep learning have been found in diagnosis and prediction across various domains [7,13,14]. Furthermore, deep learning methods notably impact classification accuracy in numerous medical tasks [15,16]. The utilization of deep neural networks (DNNs) in modern computer-aided diagnosis (CAD) systems holds the potential to reduce the efforts required for heart monitoring and improve predictive capabilities. Integrating wearable ECG monitoring systems with deep learning could further enhance the reliability of CAD systems for cardiac patients. Therefore, the IoT-based ECG monitoring system could help reduce diagnostic time and provide healthcare facilities at patients’ doorsteps.

Nevertheless, the automatic detection of heart disorders through ECG-based is faced with several important challenges, such as:

- the properties of ECG signals that, in terms of amplitude, period, etc., vary from individual to individual due to different demographic factors such as gender, age, lifestyle, etc.;
- the ECG signals of a single tested person vary across different states, such as sleeping, running, and walking;
- the noise and artifacts in the captured ECG can lead to variations and differences, as explained in the following subsection.

### Artifacts/Noises Affecting the ECG

The ECG signal can become mixed up with different types of unwanted sounds, each with its own characteristics.

- Baseline wander: This occurs when the signal changes slowly because of things like skin contact or patient movement. It adds a slow-moving section to the ECG signal that we do not want [17].
- Power line interference: This kind of noise is caused by electricity sources like power lines. It tends to show up at 50 or 60 Hz, even though we cannot always know when it will appear or how strong it will be [18].
- Motion artifacts: If the sensors move away from where they should be on the skin, it results in these unwanted changes in the signal. These represent a problem because we cannot predict how they will look or how often they will occur.
- Muscle noise: This occurs because of muscle movements and is similar to the ECG signal in terms of its energy.

To ensure consistency and accuracy in the ECG readings, it is crucial for all clinical technicians to follow the same standardized method for obtaining a 12-lead ECG. This standardization guarantees that the ECG data collected are reliable and precise. These high-quality ECG data then serve as the foundation for feeding into AI-based systems.

The AI-based system capitalizes on these reliable ECG data to carry out the analysis. Employing advanced algorithms, the system can recognize patterns, anomalies, and indicators within the ECG signals. This analysis yields valuable insights that can assist in diagnosing cardiac conditions and making informed medical decisions.

## 2. Literature Review

Digital image processing and machine learning have a significant role in healthcare. Recently, research has been concentrated on cardiac disease detection through deep learning, yielding promising results [19,20,21,22,23]. CNN is the latest method used for the classification and detection of heart signals with different variations [24,25]. Similarly, Xia et al. [26] recommended a wearable ECG detection system. The model was evaluated through ECG data acquired from a wearable patient monitoring (WPM) device and the MIT-BIH arrhythmia database. Although the authors present good classification performance, the WMP devices face accuracy, precision, and reliability issues [27]. Huang et al. [28] proposed the classification of an ECG arrhythmia using two-dimensional (2D) CNN. In this technique, the five different types of heartbeats were first converted into time–frequency spectrograms before 2D CNN was used to classify the data. The authors presented various approaches in [29], where the main focus was the application of a deep learning model with GRU (gated recurrent unit) and then the use of an ELM (extreme learning machine) for the identification of the ECG signal. Lu et al. [30] used PQRST and 2D convolutional features for the recognition of a single heartbeat, a method called the random oversampling algorithm was used to balance the data, and finally, a random forest classifier was applied. Ji et al. [31] used 1D ECG signals and transformed them into a 2D image. A faster R-CNN (faster regions with a CNN) algorithm was used for the experiment and accomplished an accuracy of 99.21%. Fan et al. [32] used a multiscaled fusion of deep CNN using a single lead short ECG and achieved an accuracy of 96.99%. Li et al. [33] suggested an e-health monitoring system of CVD by combining heartbeats into a two-dimensional feature vector and then applied CNN for efficient detection. In a research work by Naz et al. [34], deep learning techniques were proposed for the detection of cardiac disorders in ECG signals. The signals were transformed into images and then supplied into three different deep learning models: AlexNet, Inception-v3, and VGG-16. Transfer learning was applied to train all these models in the task of detecting CVD. The extracted features were combined, and the best ones were selected through a heuristic entropy method.

As most researchers used timeseries data of ECG with signal leads, which are not suitable for power line interface and muscle contraction, among other things. The most appropriate method for screening cardiac disorders usually advised by cardiologists is the use of 12-lead ECG images. The major motivation and contribution of this paper is the development of an effective automated CVD system using 12-lead ECG images for hospitals as well as for remote patients. In this work, we applied a lightweight CNN with attention module, while the results are evaluated using an accuracy, recall, precision, and confusion matrix.

The above review is summarized in Table 1.

The subsequent sections of this manuscript are organized as follows: Section 3 delineates the proposed methodology encompassing the IoT-based framework and the automated classification of ECG images. This approach involves the use of REL and DSR routing for data transformation, as well as the application of the lightweight CNN for classification. In Section 4, the results and discussion are given, and finally, the conclusions and future research directions are outlined in Section 5. 

## 3. Proposed Method

The proposed system is implemented in a two-stage approach, i.e., IoT-based ECG framework and the classification of ECG images.

### 3.1. IoT-Based ECG Framework

**Figure 2:**
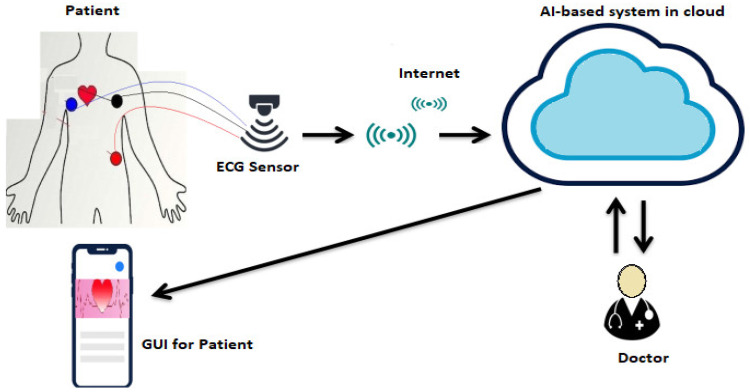
IoT-based ECG framework.

The system described primarily consists of three components: an electrocardiogram (ECG) sensing system, an internet of things (IoT) cloud, and a graphical user interface (GUI), as presented in Figure 2. The ECG sensing system often utilizes wearable sensors for continuous monitoring, which have minimal disruption to a user’s daily life. These sensors allow for the long-term recording of ECG data.

As presented in Figure 2, the IoT cloud system is composed of three main components: collection of data and transmission, ECG investigation, and disease alert. The data collection module involves the use of portable devices to collect ECG data, which are then transmitted to the cloud using a routing protocol. REL and DSR routing were employed for data transformation. The ECG analysis module is liable for extracting essential features for the identification of potential heart diseases. However, the ECG signals may be affected by noise in the data collection and communication process, which can impact the accuracy of the diagnosis. Consequently, the IoT cloud also presents data analysis architecture to extract relevant features using the ECG signals. Finally, the disease alert module is critical for protecting patients and providing an alert system to ease medical care when needed.

#### 3.1.1. DSR Protocol

The DSR protocol operates as a source-routing mechanism within the IoT cloud network, enabling the transmission of packets across multiple hops among network nodes. This is particularly useful when the distance between nodes exceeds the range of direct transmission. The protocol involves the initiation of a packet’s transmission via an originator node, which determines a specific sequence of nodes through which the packet must pass to reach its intended destination.

In the DSR protocol, when a source node (S) seeks to transmit data to a destination node (D), it first checks its route cache to determine whether a direct route to D exists. If such a route is absent, S broadcasts a route demand message to its neighboring nodes. These neighboring nodes, acting as intermediaries, either respond with route information through a route reply message if they possess a suitable route to D or they forward the request to their own neighboring nodes [35].

This iterative process continues until the route demand message reaches either D or a node that holds a viable route to D. Upon receiving a route reply message, S stores the route information in its cache, enabling subsequent data transmission along the established route.

It is important to note that the DSR protocol prioritizes route discovery only when there are data to be sent to a destination. Each node maintains a list of routes in its cache, and a node will request a route from its neighbors if it lacks a route to a specific location. The protocol’s core mechanisms include route discovery and maintenance, and while it offers simplicity and efficiency, it has limitations in handling large networks and susceptibility to specific types of routing loops and attacks.

**Figure 3:**
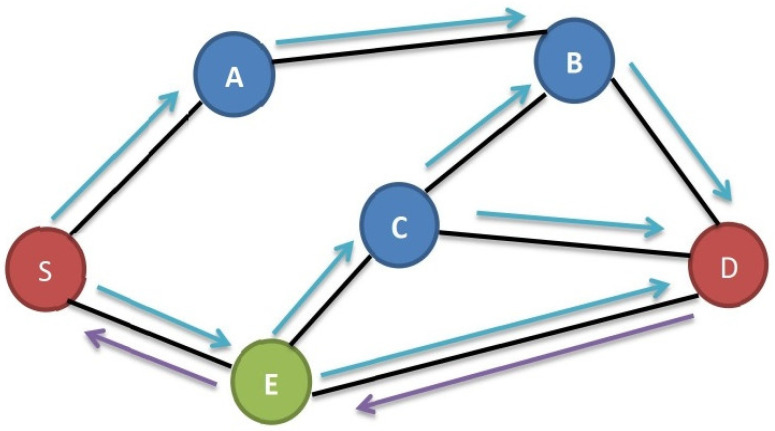
DSR protocol illustration from S to D.

Due to its characteristics, the DSR protocol is most effective in small-scale, ad hoc networks, where minimizing overhead takes precedence over scalability and robustness. The operation of the DSR protocol is summarized in Figure 3, outlining the steps from route discovery to successful data transmission, with additional nodes identified alongside the source node (S) and destination node (D) for clarity.

- The source node (S) intends to transmit data to the destination node (D).
- S verifies its route cache to determine the availability of a route to D.
- If S does not possess a route to D in its cache, it broadcasts a route demand message to its neighboring nodes.
- Each neighbor that receives the route request message checks its own route cache to see whether it has a route to D. If it does, it sends a route reply message back to S with the route information. If it does not have a route to D, it forwards the request to its own neighbors.
- This procedure carries on until the request is received by either D or a node with a route to D.
- When a route reply message is received by S, it records the route in its cache and uses it to send the data to D.

#### 3.1.2. REL Protocol

REL protocol is commonly used in wireless sensor networks (WSNs) and IoT applications such as smart cities, environmental monitoring, healthcare, and comfortable offices and homes. It uses residual energy and link quality to find routes that improve the system’s QoS (quality of service) reliability and support and incorporates an event-driven mechanism for load balancing and the prevention of early node death [36]. In WSN communication, links are often unreliable due to low-powered radios that are vulnerable to multipath distortion, noise, and interference. The effectiveness of route selection depends on the accuracy of the link quality estimate to improve reliability. Generally, a LQE (single link quality estimator) value such as LQI (link quality indicator) or RSSI (received signal strength indicator) is used to represent the link quality at a specific time, but it does not provide additional information about the end-to-end link quality, hop count, or residual energy.

The REL protocol has a number of advantages over purely reactive protocols like DSR. It has lower latency and overheads, since routes are maintained proactively rather than being discovered on an as-needed basis. It also has better scalability, since it can handle larger networks more efficiently. However, it does have some drawbacks, such as higher energy consumption and the potential for routing loops if the proactive flooding mechanism is not implemented carefully.

Thus, a hybrid approach of routing protocol is used, which combines elements of both DSR and REL. The hybrid protocol uses a REL to maintain routes to commonly used destinations, while using a DSR to discover routes to less frequently-used destinations on an as-needed basis.

### 3.2. Classification of ECG Images

**Figure 4:**
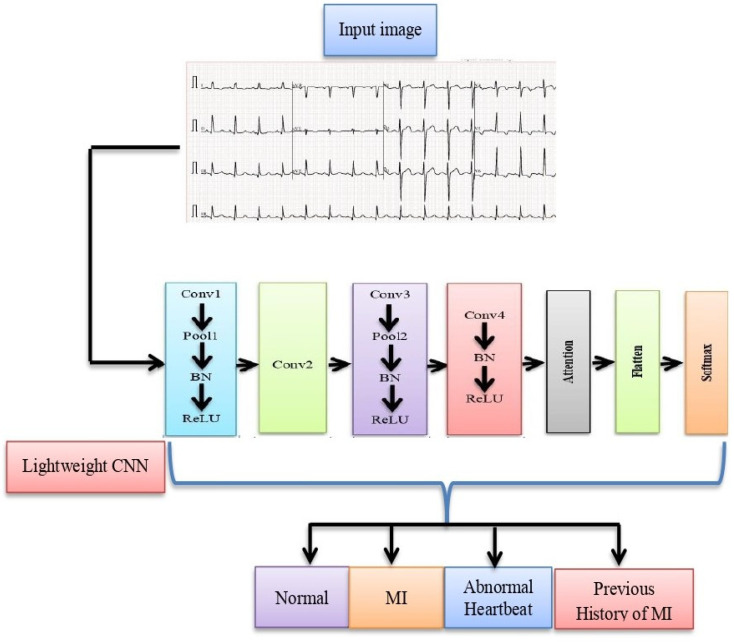
Proposed method of ECG classification.

A typical ECG consists of P and T waves, PR and ST segments, PR and QT intervals, as well as the QRS complex. The dataset employed in this work consisted of 12-lead ECG images and is publicly available [37]. All these features have been considered using the lightweight CNN model to ensure that it is equipped with the necessary information to accurately classify ECG signals into their respective categories. The details of the proposed methodology are shown in Figure 4.

#### 3.2.1. Dataset

**Figure 5:**
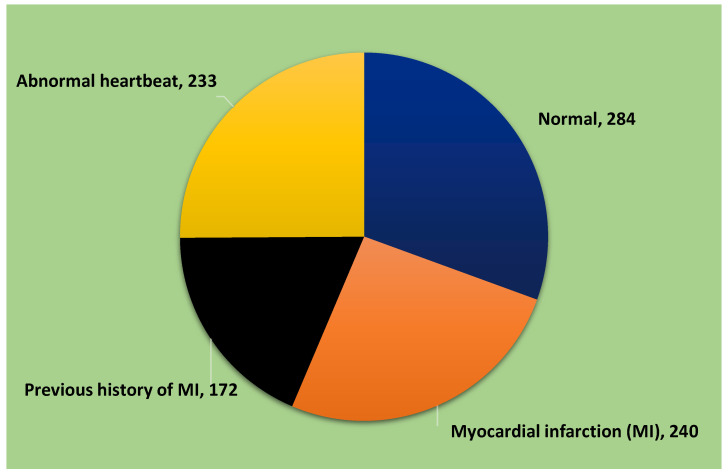
ECG data distribution.

We utilized a publicly accessible dataset in this study known as the “ECG Images dataset of Cardiac Patients” [37]. This dataset comprises 12-lead ECG images categorized into four groups: normal, myocardial infarction (MI), abnormal heartbeat, and previous history of MI. The detailed description of the 12-lead ECG Images dataset used in the proposed model is provided in Table 2, while the distribution of images within the dataset is illustrated in Figure 5.

**Figure 6:**
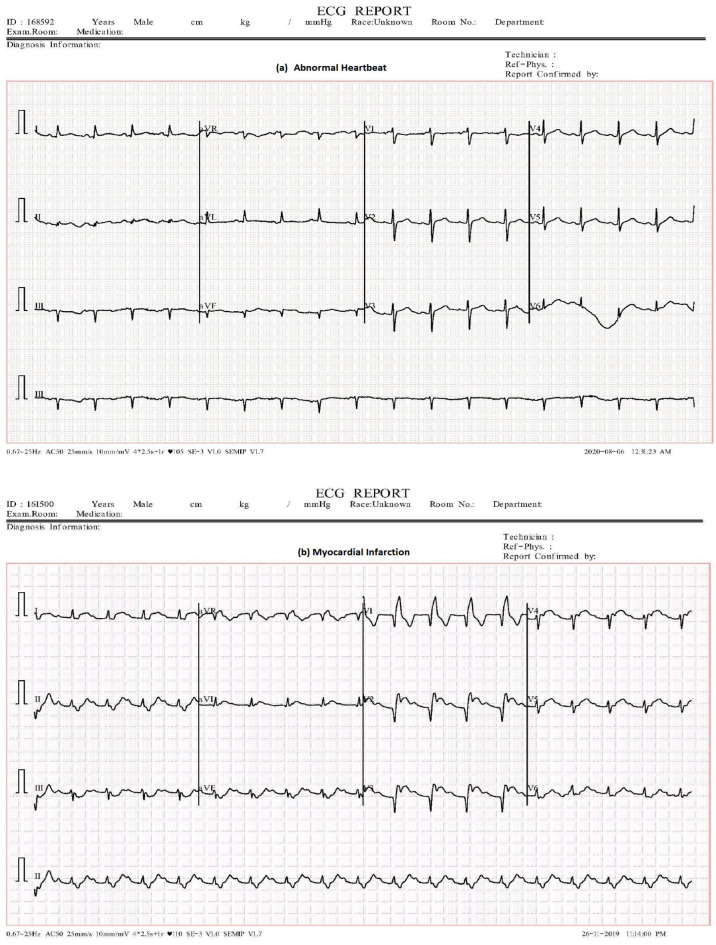
ECG samples.

To provide a clearer description of the dataset, we have chosen random sample from abnormal heartbeat and myocardial infarction (MI), as illustrated in Figure 6.

#### 3.2.2. Lightweight CNN

**Figure 7:**
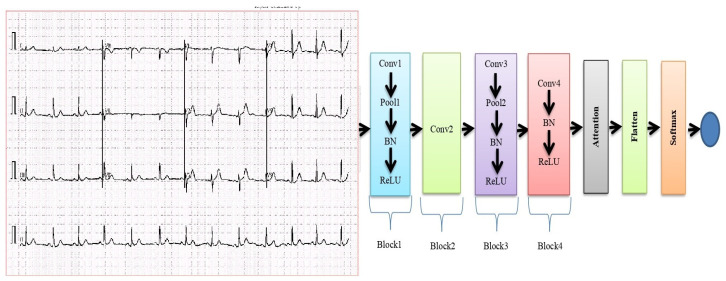
Lightweight CNN.

The proposed lightweight CNN consists of several components, as depicted in Figure 7.

##### Four-Layer Lightweight CNN

The initial component of the model is responsible for processing the input ECG data and extracting appropriate features from it. This lightweight CNN usually includes multiple convolutional layers, as well as pooling layers and activation functions. The purpose of the four-layer CNN is to effectively capture important patterns from the ECG data. The proposed lightweight CNN is employed to automatically extract ECG features. This CNN focuses on feature extraction and leaves feature optimization to the attention module, which is the method’s main focus. Additionally, the inclusion of a BatchNormalization (BN) layer and a ReLU layer in the CNN helps to improve the training process and avoid overfitting.

##### Attention Module

The attention module is a crucial component of the proposed method that enhances the characteristics appropriate to the identification category. This module is based on two primary strategies.

The contextual encoding layer: This method enables the model to discover contextual connections between various input data [38], which is helpful for identifying dependencies in sequences. By understanding contextual relationships, the model can better capture dependencies and patterns in the ECG data.

The depthwise separable convolution: This method is used to reduce the model’s number of parameters. A lighter model with fewer parameters is produced via depthwise separable convolution, which separates the spatial and channel-wise processes [39]. This makes the model more effective for use in practical applications.

##### Flattening and SoftMax Classifier

The improved features are flattened into a one-dimensional vector after going through the attention module, turning the 2D or 3D tensor into a linear array. The SoftMax classifier is then fed this one-dimensional vector. A common option for multi-class classification tasks is the SoftMax classifier, which determines the probability of each identity category based on the ECG data retrieved by the preceding layers. The input ECG sequence’s anticipated identity is the class with the highest probability.

When an ECG image is input, the model goes through three stages of the lightweight CNN model to produce a label for the image based on the identified features. The model was trained using specific parameters listed in Table 3. The loss function employed for the model is categorical cross entropy, a common choice for multiclass classification tasks. The rate of learning is set at 0.001, determining the step size of the optimizer during training. The model employs the Adam optimizer, a widely used optimization algorithm that updates model weights based on the gradient of the loss function. The training process spans 70 epochs, indicating that the complete dataset passes through the model 70 times. The dataset is divided into training and testing sets of 80% and 20%, respectively. The batch size used is 16, meaning that 16 samples are utilized to adjust model weights in each iteration of training. Finally, the activation function in the output layer is SoftMax, a standard choice for obtaining the probability distribution of expected classes in multiclass classification tasks.

The pseudo-code of the proposed system, focusing on the IoT-based ECG framework and the classification of ECG images, is described below:

- IoT-based ECG Framework
  - Initialization:
    - Initialize ECG sensing system (wearable sensors) for continuous monitoring.
    - Initialize IoT cloud for data collection, transmission, analysis, and disease alert.
  - Data Collection and Transmission:
    - Read ECG data from wearable sensors continuously.
    - Send the collected ECG data to the IoT cloud using DSR or REL routing protocol.
  - ECG Investigation:
    - IoT cloud receives the ECG data and stores them.
    - IoT cloud performs data analysis to achieve essential features using the ECG signals.
  - Disease Alert:
    - IoT cloud processes the extracted features and detects potential heart diseases.
    - If a potential heart disease is detected, the disease alert module is triggered.
    - The disease alert module sends alerts to relevant parties (medical personnel, patients) for immediate medical care.
  - Route Discovery (DSR Protocol):
    - When a source node (S) wants to send data to a destination node (D) and does not have a route in its cache:
    - S broadcasts a route request message to its neighboring nodes.
    - Each neighbor receiving the request checks its own cache for a route to D.
    - If a route is found, the neighbor sends a route reply message back to S with the route information.
    - If no route is found, the neighbor forwards the request to its own neighbors.
  - Route Maintenance (DSR Protocol):
    - When a route reply message is received by S, it records the route in its cache for future use.
    - The route is also saved in the route caches of all nodes that helped find it.
  - Route Selection (REL protocol):
    - REL protocol uses residual energy and link quality to find routes for improved QoS reliability.
    - Links are selected based on LQE values (e.g., LQI, RSSI) and residual energy.
- Classification of ECG Images
  h. Dataset Preparation:
    - Load the “ECG Images dataset of Cardiac Patients”, consisting of 12-lead-based ECG images and four classes (normal, myocardial infarction, previous history of MI, abnormal heartbeat).
    - Split the dataset.
  i. Lightweight CNN Model:
    - Input layer for processing the ECG data.
    - Multiple convolutional layers with pooling and activation functions to capture important patterns.
    - Contextual encoding layer: discover contextual connections in input data for identifying dependencies in sequences.
    - Depthwise separable convolution: reduce model parameters for efficiency.
    - Flattening: flatten the improved features into a one-dimensional vector.
    - SoftMax classifier: determine the probability of each identity category based on the extracted ECG data.

## 4. Experimentation

### 4.1. Performance Matrices

The performance of the model employed in the work was evaluated using various metrics, including the accuracy, precision, recall, and confusion matrix [40,41,42].

- Accuracy measures the proportion of correct predictions made by the model.
- Sensitivity or recall is the measure to predict true positives out of all positive
- instances.
- Precision is a measure of the ability to predict true positives from actual positive instances.
- The confusion matrix is a table that summarizes the presentation of a classifier by comparing the actual and predicted classifications.

The aim of the research was to classify various types of cardiac disorders, and the outcomes of this classification are reported in Table 4. These evaluations were carried out in order to determine how well the models were able to correctly classify the different disorders.

To classify ECG images, the following terms are used to evaluate the parameters: $T^{+}$ signifies true positive, $F^{+}$ is false positive, $T^{-}$ is true negative, and $F^{-}$ signifies false negative. The formulas of accuracy, recall, and precision are obtained based on Table 4, and are given as Equations (1)–(3).

(1) $$\mathrm{Accuracy}=\frac{\left(T^{+}+T^{-}\right)}{\left(T^{+}+F^{-}\right)+\left(F^{+}+T^{-}\right)}$$

(2) $$\mathrm{Sensitivity}\;\mathrm{or}\;\mathrm{Recall}=\frac{\left(T^{+}\right)}{\left(T^{+}+F^{-}\right)}$$

(3) $$\mathrm{Precision}=\frac{\left(T^{+}\right)}{\left(T^{+}+F^{+}\right)}$$

### 4.2. Results and Discussion

The description of the lightweight model’s parameters is depicted in Table 5. The Table provides a comprehensive summary of the model’s architecture, including the number of float operations and the input shape for each layer.

The proposed lightweight CNN model has achieved an impressive accuracy with the possibility of further enhancement through the fine-tuning of hyper-parameters. The results in Table 6 display the performance of the model. The accuracy, which is 98.39%, represents the portion of correct predictions. Precision, given as 0.985, indicates that about 98.5% of the positive predictions made by the model were accurate. Recall, at 0.98, signifies that the model identified around 98% of all actual positive cases.

**Figure 8:**
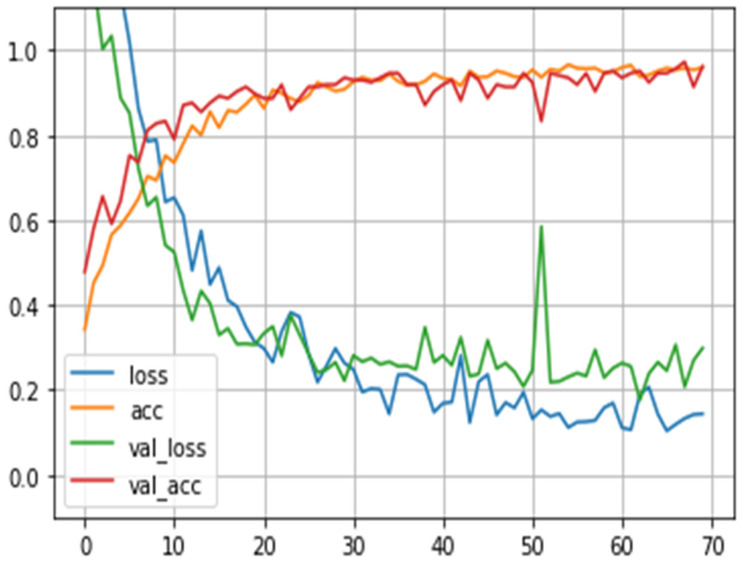
Loss vs. accuracy.

**Figure 9:**
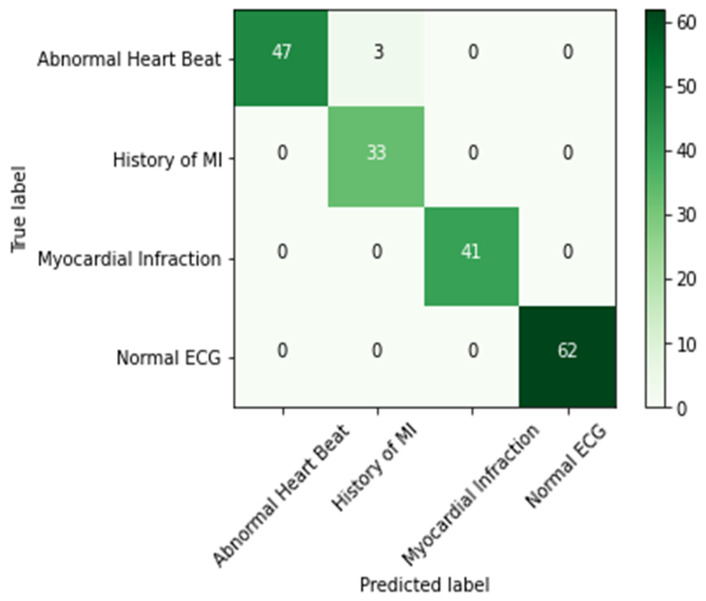
Confusion matrix.

The results are presented graphically in Figure 8, illustrating the loss and accuracy trends across each epoch during the training phase. The graph distinctly illustrates that the model’s accuracy improved with the progression of training epochs. This outcome gains further validation through the utilization of a confusion matrix (Figure 9). This matrix offers a concise overview of how well the model performed in terms of its projected and actual classifications. Notably, it is important to mention that the confusion matrix displayed in Figure 9 is derived exclusively from the test data, constituting 20% of the total dataset.

Clearly, these results show that the model’s ability to comprehend hierarchical features from a limited dataset underscores its potential to capture intricate patterns and representations within the data, resulting in enhanced performance in tasks characterized by data scarcity. Furthermore, its deliberate design prioritizes computational efficiency, distinguishing it from deeper and more intricate CNN architectures. This efficiency renders the lightweight CNN particularly suitable for resource-constrained scenarios, such as IoT devices and wearable sensors, where computational resources may be restricted. The advantage of a lightweight CNN lies in its automatic feature learning capability from the raw ECG data. By directly capturing abstract patterns and representations from the data, it enhances the potential for better discrimination between different classes. This feature extraction process removes the requirement for manual feature engineering, simplifying the model’s architecture, and contributing to its overall interpretability.

**Figure 10:**
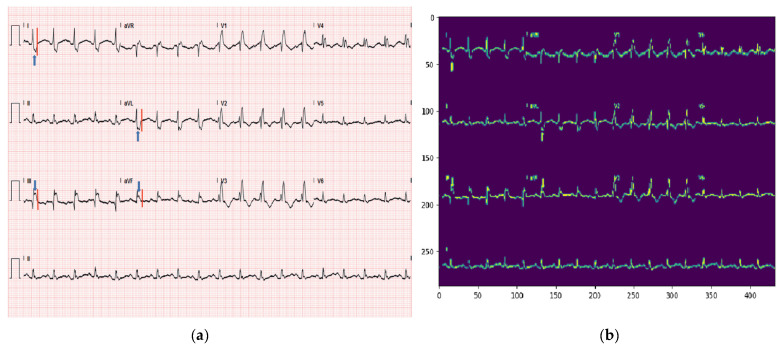
(**a**) Input image of MI. (**b**) Predicted image as MI.

This manuscript discusses an IoT-based cardiac patient monitoring and detection system, which, by design, involves real-time data collection from IoT sensors. However, to demonstrate the model’s effectiveness, we initially used a publicly available ECG image dataset, widely accepted as a benchmark in the field. This dataset served as an initial testbed, and its results are presented in Figure 10, where an input image is successfully predicted. We specifically worked with a dataset consisting of 12-lead cardiac ECG images recorded at a 500 Hz frequency. However, we acknowledge that ECG images can significantly vary across different sources and clinical settings. To ensure our model’s robustness and applicability to a wider range of datasets, including real-world data, extensive training is necessary.

Finally, Table 7 presents a comparison between the outcomes of our proposed method and the models that use the same dataset for classifying ECG images. Through an extensive search, we found that the only existing studies utilizing the same dataset for classifying the four classes are [5,43]. Our proposed lightweight CNN method has clearly demonstrated its excellence by achieving superior accuracy and precision, thereby establishing its effectiveness in ECG image classification.

## 5. Conclusions and Future Work

This paper presents a solution aimed at enhancing the management of a CVD through early diagnosis. Despite the advancements in clinical treatment, the accurate detection of cardiac disorders remains a challenging task. Deep learning has emerged as a promising approach to improve diagnosis, especially in remote patient scenarios, achieving an impressive accuracy of 98.39%. However, a limitation of this research is the absence of the segmentation process, which could have increased computational complexity and further improved the results.

Incorporating the segmentation approach before classification is the work’s future goal because it is anticipated to improve the diagnostic process’ accuracy and dependability. The suggested solution intends to further improve and streamline the CVD detection process by utilizing robust features and investigating alternative deep learning models on more datasets. These initiatives could change early detection and open the door to the better management of heart diseases.

## Figures and Tables

**Table 1:** Literature review conclusions.

Research Work	Method	Dataset	Results
Imran et al., 2022 [14]	Wearable ECG detection system	ECG data from wearable patient monitoring device and MIT-BIH arrhythmia database	Claimed good classification performance
Chamatidis et al., 2017 [21]	Faster R-CNN	-	99.21% accuracy in detecting ECG signals
Isin et al., 2017 [22]	Multiscaled fusion of deep CNN	Single lead short ECG	96.99% accuracy
Naz et al., 2021 [34]	Deep learning techniques (AlexNet, Inception-v3, VGG-16) + transfer learning	-	Claimed good results

**Table 2:** Image distributions based on classes.

S/No.	Activities
Normal	284
Myocardial infarction (MI)	240
Previous history of MI	172
Abnormal heartbeat	233

**Table 3:** Proposed parameters for lightweight CNN.

Name	Description
Batch size	16
Optimizer	Adam
Training	80%
Loss Function	Categorical cross-entropy
Epochs	70
Learning rate	0.001
Testing	20%
Activation	SoftMax

**Table 4:** Prediction outcomes.

Outcome	Definition
$T^{-}$	Correct identification of negative data
$T^{+}$	Correct identification of positive data
$F^{-}$	Incorrect identification of negative data
$F^{+}$	Incorrect identification of positive data

**Table 5:** Float operations.

Layer	Float Operations	Input Shape
Conv2D	7,962,624	1, 288, 432, 1
Conv2D	7,077,888	1, 24, 72, 32
MatMul	786,432	1, 3072
BiasAdd	442,368	1, 96, 144, 32
MaxPool	442,368	1, 96, 144, 32
BiasAdd	12,288	1, 8, 24, 64
MaxPool	12,288	1, 8, 24, 64
MatMul	1024	1, 128
BiasAdd	128	1, 128
SoftMax	20	1, 4
BiasAdd	4	1, 4
All Layers	16,737,432	

**Table 6:** Obtained results.

Accuracy (%)	Precision	Recall
98.39	0.985	0.98

**Table 7:** Comparison with recent techniques.

Refs.	Year	Method	Dataset	Accuracy (%)	Recall (%)	Precision (%)
[5]	2021	MobileNet v2	ECG images for Cardiac patients	97.5	-	-
[43]	2022	CNN		98.23	98.22	98.31
Proposed	-	Lightweight CNN		98.39	98	98.5

## Data Availability

The dataset used in this research is available online at https://data.mendeley.com/datasets/gwbz3fsgp8/2 (accessed on 20 July 2023).
